# Development of a 3D nnU-Net-based cell tracking platform for quantifying myocardial deformation in zebrafish

**DOI:** 10.1016/j.isci.2026.116376

**Published:** 2026-06-18

**Authors:** Tanveer Teranikar, Mishu Devadasan, The Van Le, Yoonsuk Kang, Gilberto Hernandez, Phuc Nguyen, Yichen Ding, Cheng-Jen Chuong, Jin Young Lee, Hyunsuk Ko, Juhyun Lee

**Affiliations:** 1 Joint Department of Bioengineering at the University of Texas, Arlington, TX, USA; 2 Department of Artificial Intelligence and Robotics, Sejong University, Seoul, South Korea; 3 Department of Bioengineering, University of Texas, Dallas, TX, USA; 4 Department of Electrical Engineering, Hanyang University, Seoul, South Korea; 5 Department of Electrical Engineering, DGIST (Daegu Gyeongbuk Institute of Science and Technology), Daegu, South Korea

**Keywords:** Cell biology, Bioinformatics

## Abstract

Quantifying three-dimensional preclinical myocardial deformation *in vivo* currently remains a challenging task, as non-invasive cardiac imaging methods are often restricted to two-dimensional projections suffering from limited reproducibility. Here, we introduce a 4D (3D + time) imaging and analysis platform that integrates a benchtop high-frame rate, modular light-sheet microscope (LSM) integrated with a deep-learning-enabled cardiomyocyte tracking pipeline. Our LSM achieves cellular resolution up to millimeter-scale field-of-view (FOV) at millisecond temporal sampling, allowing reconstruction of 4D trajectories of individual cardiomyocyte nuclei within embryonic zebrafish. To address segmentation failures arising from spatially overlapping nuclei within multilayered myocardium, we incorporated a difference-of-Gaussian and watershed-based preprocessing module into a 3D nnU-Net framework, enabling robust separation of cell-cell contact and mitigating nucleus-merging artifacts. This workflow supports reproducible extraction of myocardial deformation metrics and provides a scalable foundation for disease-specific biomechanical modeling. Together, our approach demonstrates how combining advanced LSM instrumentation with deep-learning-driven reconstruction pipelines can accelerate autonomous, high-throughput imaging of cardiac microstructure *in vivo*.

## Highlights

• Integrated 4D microscopy with deep learning platform for tracking cell trajectories
• Implemented watershed segmentation for multidimensional feature detection
• Validated nnU-Net with particle tracking algorithms for high-throughput cell analysis
• Zebrafish myocardial analysis exhibited regionally anisotropic ventricular strain

## Graphical abstract

**Figure:**
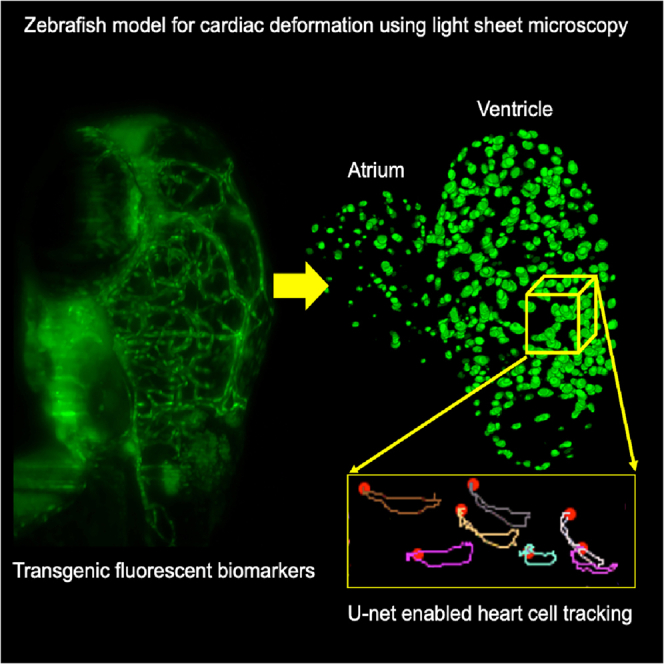

## Introduction

Investigating genetic, structural, or metabolic biophysical characteristics in response to cell dysfunction or targeted therapeutic intervention is fundamental to biomedical research.^1^^,^^2^^,^^3^ Advances in computational biomedical technologies over the past decade have significantly enhanced the understanding of cell-associated genomics, disease precursors, and imaging biomarkers, thereby, facilitating disease modeling and the clinical translational of pharmaceuticals.^4^^,^^5^^,^^6^ However, linking molecular perturbations to real-time biomechanical behavior at the single-cell level *in vivo* remains technically limited, particularly in rapidly developing organs such as the embryonic heart.

Among non-communicable diseases, cardiovascular disease (CVD) and hemodynamic malformations are leading causes of death in the 21st century.^7^ CVD research demands a comprehensive understanding of cardiac cell lineages and associated phenotypes, arising from tissue remodeling driven by hemodynamic malformations.^8^ Consequently, identification of novel hemodynamic biomarkers to quantify local tissue deformation and cardiac output has become a crucial research focus. Yet, current imaging and computational approaches fall short of capturing these biomechanical phenomena at cellular resolution across an entire cardiac cycle.

The human heart functions as a dynamic pump, subjected to mechanical stimuli across an entire cardiac cycle.^9^^,^^10^^,^^11^^,^^12^^,^^13^^,^^14^^,^^15^ Previous translational studies in zebrafish indicate that healthy cardiomyocytes (CM) responsible for contractile motion, exhibit anisotropic mechanical forces according to their location within the heart.^11^^,^^12^^,^^13^^,^^14^^,^^15^ Hence, understanding the complex mechanotransduction of cardiac tissue requires diagnostic platforms capable of multidimensional, *in vivo* cell tracking within animal models. In this regard, zebrafish have emerged as robust animal models for cardiac cell tracking or chemical screening of pharmaceuticals. This is attributed to the optical clarity of the specimen, low-cost maintenance, and high reproducibility of transgenesis.^16^^,^^17^^,^^18^^,^^19^ Moreover, homology between genetic and signaling pathways of zebrafish and humans, enhances their relevance for cardiovascular research. However, visualizing dynamic zebrafish cardiac cells is challenging due to their small size (100–150 μm) and rapid motion (∼170 bpm).^11^ As a result, transgenic zebrafish studies have required the evolution of advanced imaging platforms, capable of providing high spatiotemporal resolution for cell tracking.

In this regard, light sheet microscopy (LSM) is a popular fluorescence modality among zebrafish biologists.^20^ LSM can provide cellular resolution with reduced phototoxicity, while maintaining rapid 3D volume acquisition.^21^ In addition, LSM offers promising avenues for autonomous microscopy in combination with semantic segmentation networks requiring extensive training data.^22^ On the other hand, the LSM modality intrinsically suffers from background noise due to planar illumination, adversely affecting the contrast of overlapping features and resulting in data sparsity along cross-sectional views.^21^^,^^23^ Hence, conventional intensity thresholding techniques or deep learning methods such as convolutional neural networks (CNNs), inaccurately label features in high dimensional image space. Such algorithms are unable to deliver scalable segmentation performance in the presence of overlapping textures and require manual ROI annotation,^24^^,^^25^ affecting time-lapse tracking of annotated regions.^26^^,^^27^ Therefore, an automated, generalizable segmentation framework capable of isolating overlapping nuclei and supporting 4D tracking is essential for *in vivo* cardiac biomechanics.

To address these unmet challenges, nnU-Net has recently emerged as a robust, self-configuring tool for medical image segmentation. Unlike traditional deep-learning methods that require extensive manual tuning, nnU-Net autonomously optimizes network configurations based on domain-specific data.^28^^,^^29^ The application of instance normalization within computational outputs, enhances segmentation performance across diverse imaging modalities.^30^ Nevertheless, its application to high-speed embryonic cardiac imaging—where nuclei overlap spatially, deform dynamically, and move rapidly—remains largely unexplored.

In this study, we implemented a zebrafish-specific 3D nnU-Net architecture, to enable a high-throughput, automated workflow for annotation of 3D + time myocardial nuclei. This article is partly based on chapters 3 and 6 of the author’s PhD thesis and includes methodological developments beyond the original thesis.^31^ In this regard, we have integrated a difference-of-Gaussian (DoG) and watershed preprocessing framework within 3D nnU-Net. Hence, cell merging events and overlapping features were successfully delineated without any manual intervention, resulting in no significant difference compared with manually annotated ROI. By combining 3D nnU-Net-based segmentation with rapid 4D LSM imaging, we reconstructed full cardiac-cycle nuclear trajectories and quantified myocardial area displacement across 4–6 days post-fertilization (dpf). Together, these advances establish an integrated imaging and computational framework for scalable, autonomous, and high-fidelity mechanobiological quantification of developing myocardium.

## Results

### Zebrafish CM-specific nnU-Net architecture

**Figure 1:**
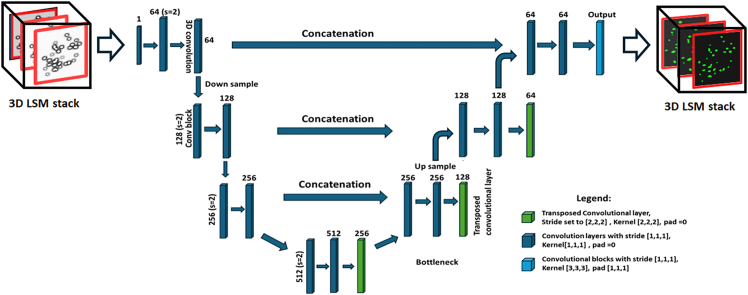
Zebrafish nnU-Net architecture The network is composed of convolutional blocks, each containing a convolutional layer followed by instance normalization and a leaky ReLU activation. Block numbers indicate the corresponding output feature-map size. Downsampling is performed using strided convolutions, while upsampling is achieved using transposed convolutions.

The nnU-Net is a supervised, self-configuring network that demonstrates robust generalization of segmentation pipeline parameters for varying datasets without requiring manual configuration. The nnU-Net architecture employs a U-Net-based architecture with two computational blocks per resolution stage in both the encoder and decoder paths. Each computational block consists of convolution, instance normalization, and leaky RELU (Figure 1). The decoder convolutional blocks mirror those of encoder, with down-sampling performed using stride convolutions (stride = 1 × 2 × 2 or 2 × 2 × 2), while up-sampling is implemented using transposed convolutions. A distinctive feature of nnU-Net is its rule-based configuration approach, which establishes interdependencies between image metadata (voxel spacing and median image size) and pipeline configuration (patch size, batch size, GPU memory constraints). This design allows the network’s fixed U-Net based architecture to iteratively optimize larger batch sizes, while minimizing GPU memory usage.

On the other hand, instance normalization of features leads to inaccurate pixel classification for overlapping nuclei distributed across varying tissue depth. This issue arises because instance normalization inadvertently transfers outlier’s (background noise) “style” to nnU-Net content (Table 1), during activation of convolutional outputs at each instance. Taking advantage of this style transfer, we integrated DoG edge detection and watershed cell splitting within nnU-Net preprocessing. Thereby implementing a cell splitting framework within nnU-Net, by translating the style of conventional image processing algorithms. Therefore, enabling background and foreground texture information is crucial for isolating merged nuclei of varying scales.

**Table 1:** Computation performance and metrics testing

Hardware specifications		
Component	Specification	Details
GPU	NVIDIA GeForce RTX 4090	24 GB VRAM, CUDA 12.9, Driver 576.83
CPU	Intel Core i9-14900 K	24 cores (8 P+16 E), 3.2–6.0 GHz
RAM	64 GB DDR5	system memory
Storage	NVMe SSD	recommended for I/O performance
OS	Ubuntu 24.04 LTS	WSL2 environment

Training Performance			
Configuration	Time	GPU memory	Details
nnU-Net (per day)	12.0 h	18–20 GB	1,000 epochs @ 43 s/epoch
nnU-Net (all 4 days)	48 h	18–20 GB	one-time training cost
SwinUNETR (per day)	6.2 h	0.19 GB	1,000 epochs @ 22 s/epoch

Inference Performance (nnU-Net)			
Task	Time	GPU memory	Details
Per 3D volume (512 × 512×184)	69 s	6–8 GB	single case
Throughput	52 cases/h	6–8 GB	continuous processing
Cross-day evaluation	6.7 h	6–8 GB	16 models, ∼350 cases

Complete Pipeline Performance			
Component	Time	Memory	Details
Preprocessing (per volume)	5–10 s∗	4 GB RAM	DoG + watershed
Segmentation (per volume)	69 s	6–8 GB GPU	nnU-Net inference
Tracking (per specimen)	10–15 min∗	8 GB RAM	LAP tracking (linear assignment)
End-to-end (per specimen)	45–60 min∗	20 GB GPU +12 GB RAM	raw images → tracked cells

Expected Performance Scaling (Alternative Hardware)			
GPU model	VRAM	Expected time/volume	Notes
RTX 3080	10 GB	∼90–100 s	limited by VRAM
RTX 3090	24 GB	∼75–80 s	full volume processing
RTX 4090	24 GB	∼69 s	used in this study
A100	40 GB	∼50–55 s	data center deployment

### Reconstruction of dynamic cell trajectories for *in vivo* area ratio analysis

**Figure 2:**
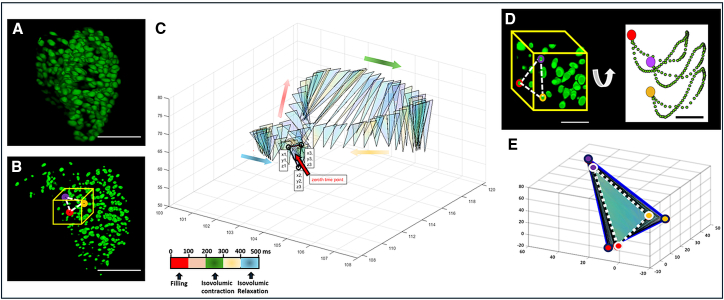
Quantifying *in vivo* mechanical deformation using local area ratio derived from cardiomyocyte nuclei trajectories (A) Cardiomyocyte nuclei in a 4 dpf embryonic zebrafish ventricle from LSM (scale bars, 40 μm). (B) Binarized segmented labels generated by the 3D nnU-Net pipeline. Yellow box: nuclei triad selected for area-ratio analysis, shown in separate colors (scale bars, 40 μm). (C) Mechanical deformation plotted across the cardiac cycle as a triangular vector patch formed by the nuclei triad in the global coordinate system. (D) Leveraging the multi-scale imaging capability of LSM, individual nuclear trajectories were tracked across a millimeter-scale field of view (white scale bars, 30 μm; black scale bars, 10 μm). (E) To compare tissue deformation, the nuclei triad was reoriented from the global coordinate system into a local coordinate frame, enabling visualization of area changes from the same perspective. In the area-ratio computation, the dotted triangle represents the systolic phase, while the solid blue boundary corresponds to the diastolic phase.

Using non-gated, 4D LSM, we acquired time-dependent ventricular myocardial deformation of *Tg(cmlc:GFPnuc)* zebrafish at distinct embryonic developmental stages (Figure 2A). The combination of LSM and the customized 3D zebrafish nnU-Net forms a robust, autonomous platform for imaging-based cell tracking. The imaging workflow leverages fully automated GUI-based 4D image reconstruction using LSM and self-configuring network architecture of the 3D zebrafish nnU-Net, enabling automated feature detection (Figure 2B**)**. We integrated the multiscale imaging capability of LSM, which allows for efficient acquisition of cellular details while maintaining a broad field of view. Simultaneously, the ability of nnU-Net facilitated the process of large patch and batch sizes without exceeding GPU memory constraints. The integrated approach enabled the acquisition and analysis of individual nuclei trajectories (15–20 μm), covering the entire ventricular circumference (50–100 μm). To reconstruct periodic deformation, we performed post hoc plotting of nuclei labels over a complete cardiac cycle (Figures 2C and 2D; Figures S1A–S1C**)**. To validate volumetric reconstruction for single nuclei, we quantified lateral resolution of 4.25 μm and axial resolution of 9.75 μm, by acquiring single micrometer beads embedded within agarose^31^ (Figures S2A–S2C). To minimize rotation and scaling artifacts, we transformed the global coordinate system into local coordinates using a geometric center as a reference. The transformation ensures that changes in the reconstructed trajectories are reflective of biological motion rather than image alignment artifacts (Figure 2E).

### Zebrafish myocardial deformation analysis using area ratio as surrogate biomarker

**Figure 3:**
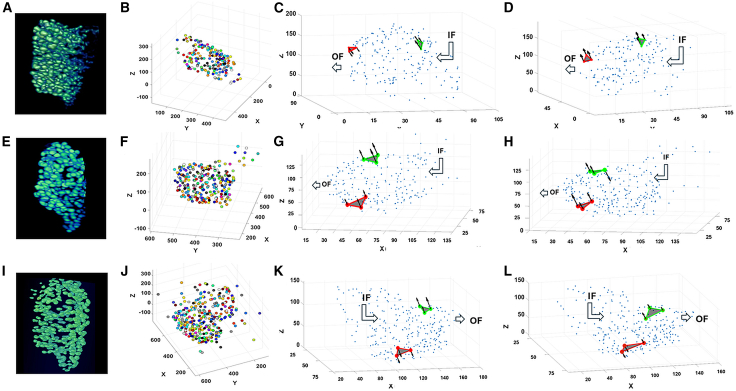
Visualization of *in vivo* mechanical deformation using local area ratio across developmental stages (A) Grayscale 3D reconstruction of ventricular cardiomyocyte nuclei at 4 dpf acquired by LSM. (B) Binarized nuclear segmentation generated by the 3D nnU-Net pipeline in the global coordinate frame. (C and D) Nuclear centroids transformed into a local coordinate frame across diastolic and systolic phases to enable rotation-invariant area-ratio analysis. (E) Grayscale 3D reconstruction of ventricular cardiomyocyte nuclei at 5 dpf acquired by LSM. (F) Binarized nuclear segmentation generated by the 3D nnU-Net pipeline at 5 dpf. (G and H) Nuclear centroids transformed into the local coordinate frame at 5 dpf across diastolic and systolic phases. (I) Grayscale 3D reconstruction of ventricular cardiomyocyte nuclei at 6 dpf acquired by LSM. (J) Binarized nuclear segmentation generated by the 3D nnU-Net pipeline at 6 dpf. (K and L) Nuclear centroids transformed into the local coordinate frame at 6 dpf across diastolic and systolic phases, enabling consistent area-ratio comparison despite heart torsion.

To validate generalization of our deep learning-based segmentation platform, we captured 4D (3D + time) ventricular deformation of transgenic zebrafish at distinct developmental stages (Figure 3) (Videos S1 and S2). Consequently, our datasets consisted of images with varying signal-to-noise ratio due to rapid camera frame rates (30–50 ms). In addition to merged nuclei clusters aggravating localization of individual cells. Hence, we integrated DoG and watershed-based preprocessing within our deep learning segmentation model, to isolate clustered nuclei. As a result, we successfully implemented area ratio as a surrogate measure for assessment of ventricular deformation, by forming a triangular region between three neighboring fluorescent myocardial nuclei. More importantly, our segmentation model enabled study of nuclei trajectories across an entire cardiac cycle from end diastolic to isovolumic relaxation phase (Figures 3A–3L) without any image registration or manual tracking.

Video S1. 4D myocardial nuclei at 4 dpf across cardiac cycle 4D (3D + time) myocardial nuclei in zebrafish ventricle captured across entire cardiac cycle at 4 dpf (related to Figure 3)

Video S2. 4 dpf nuclei binarized across cardiac cycle 4 dpf nuclei in zebrafish ventricular myocardium binarized across cardiac cycle (related to Figure 3)

### Zebrafish nnU-Net for measuring local cardiac tissue deformation

**Figure 4:**
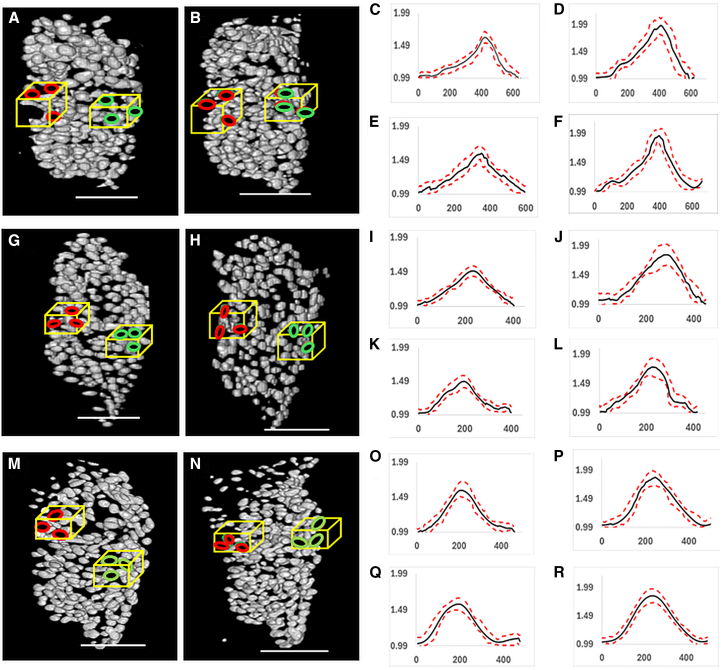
Deformation analysis of the embryonic zebrafish ventricle across distinct stages of heart maturation using nnU-Net (A and B) show dynamic ventricular myocardial nuclei binarized by 3D nnU-Net during end-diastolic filling and isovolumic relaxation at 4 dpf. (C and D) show local area deformation (area ratio) quantified using nuclei binarized by DoG and watershed without deep-learning assistance in the ventricular inner and outer regions, while (E) and (F) show area ratios derived from 3D nnU-Net segmentation in the inflow and outflow regions at the same stage. (G and H) show myocardial nuclei binarized by 3D nnU-Net during diastolic and systolic phases at 5 dpf. (I and J) show ground-truth area ratios computed using DoG and watershed in the ventricular inflow and outflow regions at 5 dpf, whereas (K and L) show the corresponding area ratios computed using nuclei binarized by 3D nnU-Net. (M and N) show diastolic and systolic nuclei deformation reconstructions at 6 dpf using 3D nnU-Net. (O and P) show area ratios calculated using ground-truth binarized nuclei in the inner and outer ventricular regions, with the corresponding nnU-Net-based area ratios shown in (Q) and (R). Solid red outlines indicate nuclei in the outer ventricular bulge, whereas solid green outlines indicate nuclei in the inner region near the inflow. Scale bars, 40 μm.

Using the zebrafish 3D nnU-Net, we segmented zebrafish CMs from the ventricular myocardium from 4 to 6 dpf (Figure 4) (Videos S3, S4, S5, S6, and S7), across entire cardiac cycles. We tracked the relative movements of nuclei triads in both the inner curvature of the ventricle near the inflow and the outer curvature of ventricular regions to analyze regional deformation of the ventricular myocardium.

Video S3. Real-time nuclei trajectories at 3 dpf Visualization real time 3D + time nuclei trajectories within embryonic zebrafish heart at 3 dpf from end-diastole to end-systole Each colored line represents the displacement path of an individual cardiomyocyte during the cardiac cycle, (related to Figure 4)

Video S4. 4 dpf hemodynamic contractility 3D + time reconstruction of dynamic myocardial nuclei used to quantify hemodynamic contractility of the zebrafish ventricle at 4 days post-fertilization Green nuclei represent nuclei in the inner region near inflow, and red centroids indicate outer ventricular region (related to Figure 4)

Video S5. 5 dpf hemodynamic contractility 3D + time reconstruction of dynamic myocardial nuclei used to quantify hemodynamic contractility of the zebrafish ventricle at 5 days post-fertilization Green nuclei represent nuclei in the inner region near inflow, and red centroids indicate outer ventricular region (related to Figure 4)

Video S6. 6 dpf hemodynamic contractility 3D + time reconstruction of dynamic myocardial nuclei used to quantify hemodynamic contractility of the zebrafish ventricle at 6 days post-fertilization Green nuclei represent nuclei in the inner region near inflow, and red centroids indicate outer ventricular region (related to Figure 4)

Video S7. displacement vectors for area ratio 3D + time reconstruction of displacement vectors of dynamic nuclei triad used for area ratio quantification The length of arrows indicates the magnitude of force acting on individual nuclei and the direction indicates displacement of nuclei during cardiac cycle (related to Figure 4)

At 4 dpf (Figures 4A and 4B), the AR values were 1.61 ± 0.09 near inflow and 1.97 ± 0.2 at ventricular outflow (Figures 4C and 4D) for non-deep learning segmented nuclei, in contrast to 1.59 ± 0.13 (*p* = 0.09, one-way ANOVA) and at 1.95 ± 0.2 (*p* = 0.18) produced by 3D nnU-Net with DoG (Figures 4E and 4F). At 5 dpf (Figures 4G and 4H), we quantified 1.58 ± 0.12 and 1.86 ± 0.12 using DoG near inner curvature and outflow regions (Figures 4I and 4J), with respect to 1.56 ± 0.09 (*p* = 0.19) and 1.84 ± 0.19 (*p* = 0.19) (Figures 4K and 4L) produced by 3D nnU-Net across inflow and outflow regions, respectively. Moreover at 6 dpf (Figures 4M and 4N), we quantified 1.50 ± 0.07 and 1.83 ± 0.19 (Figures 4O and 4P) using non-deep learning segmentation in ventricular inflow and outflow, as compared to 1.48 ± 0.09 (*p* = 0.18) and 1.81 ± 0.16 (*p* = 0.065) for 3D nnU-Net (Figures 4Q and 4R).

These results demonstrate that our 3D nnU-Net pipeline accurately quantified regional myocardial wall deformation during zebrafish development. Notably, the outer curvature consistently exhibits higher contractility than the inner curvature, suggesting that myocardial deformation varies regionally during cardiac development. Furthermore, decreasing area ratio trends indicate development with increasing mechanical efficiency with regards to heart muscle maturation.

### Validating segmentation accuracy of 3D nnU-Net with DoG preprocessing

For understating nnU-Net accuracy with respect to commonly used U-Net based deep learning models, we quantified dice coefficients for 3D myocardial nuclei segmentation across different heart developmental stages in transgenic zebrafish. 3D nnU-Net, 3Dee cell tracker using watershed post-processing without DoG and transformer-based 3D Swin U-Net were used in this validation study. In addition, we also conducted nuclei count studies to validate model robustness with regards to varying cell density, SNR, and sample movement.

**Figure 5:**
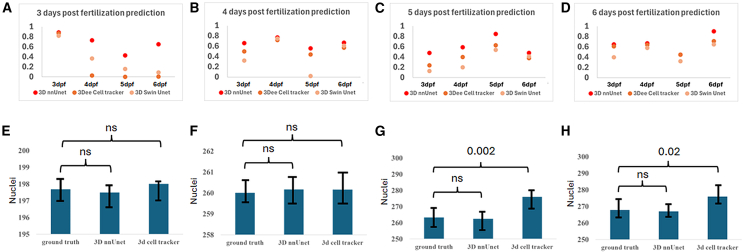
Validation metrics for nnU-Net prediction in comparison with state-of-the-art models (A–D) Dice coefficient scores quantified by testing the 3D nnU-Net model, using preprocessing based on DoG edge detection and watershed filtering, against three benchmark segmentation approaches: a 3D U-Net-based deep learning model using a feedforward network (FFN), the watershed-based 3Dee cell tracker, and the transformer-based 3D Swin U-Net. Segmentation performance for each model was evaluated at multiple developmental stages, and the corresponding Dice scores are reported. (E–H) 3D nuclei count comparison between DoG manual annotation, 3Dee cell tracker and 3D nnU-Net. ns, non-significant; one-way Anova *p* value = 0.05.

We quantified highest dice scores of 0.88 for 3 dpf, 0.77 for 4 dpf, 0.85 for 5 dpf, and 0.9 for 6 dpf for 3D nnU-Net with DoG preprocessing and watershed (Figures 5A–5D). 3Dee cell tracker, a novel deep learning-based 3D U-Net model with FFN, that was developed for 3D + time cell tracking in zebrafish, achieved comparable dice scores 0.87 for 3 dpf, 0.72 for 4 dpf, 0.63 for 5 dpf, and 0.71 for 6dpf. Notably, 3Dee cell tracker also uses watershed in post-processing with a self-learning architecture, like 3D nnU-Net. Lastly, transformer-based 3D Swin U-Net reported dice scores of 0.82 for 3 dpf, 0.75 for 4 dpf, 0.54 for 5 dpf, and 0.65 for 6 dpf (Figures 5A–5D). Hence, we quantified robust segmentation performance of DoG edge detection in combination with watershed for 3D nnU-Net.

Furthermore, we have conducted 3D cell count analysis between 3Dee cell tracker based on 3D U-Net architecture with watershed post-processing, 3D nnU-Net with DoG and watershed post-processing and ground truth manually annotated data using DoG with watershed in a previous study.^31^ The study was conducted on zebrafish ventricular volumes at varying stages of heart development, with matured heart stages (>96 dpf) containing dense nuclei clusters. While the study did not report any inter-observer reliability, the task of manual annotation was assigned to a single research scientist.

Consequently, we observed no significant differences in 3D nuclei volumes at 3 dpf between ground truth (nuclei = 197 ± 2), and 3D nnU-Net (nuclei = 197 ± 3, *p* value = 0.4) and 3Dee cell (nuclei = 198 ± 3, *p* value > 0.05) (Figure 5E). Similarly, no significant difference was observed at 4 dpf, between ground truth DoG annotation (nuclei = 260 ± 2), 3D nnU-Net (nuclei = 260 ± 3, *p* value > 0.05), and 3Dee cell tracker (nuclei = 260 ± 2, *p* value > 0.05) (Figure 5F). Notably, at 5 and 6 dpf we observed significant over segmentation by 3Dee cell tracker, due to lack of edge detection between neighboring cell clusters. At 5 dpf, we quantified ground truth and 3D nnU-Net nuclei count as 263 ± 5 for manually annotated nuclei versus 262 ± 9 nuclei for 3D nnU-Net (*p* value > 0.05). However, post hoc comparison using Tukey for 3Dee cell tracker produced 275 ± 9 nuclei (*p* value < 0.002) (Figure 5G) with respect to control group. We quantified differences in volume count at 6 dpf as well between ground truth manual annotation (nuclei = 267 ± 5) and 3Dee cell tracker (275 ± 7, *p* value = 0.02, Tukey HSD) (Figure 5H). In this regard, we hypothesize integration of DoG edge detection aided object detection in clumped cell clusters due to foreground isolation, in addition to watershed filter for cell splitting.

Lastly, we conducted cross-day validation for 3D nnU-Net and 3D SwinUNETR models for 1,000 epochs under identical training conditions (Table 2). 3D nnU-Net consistently achieved >90% Dice scores for full resolution (58,860 slices) and reduced datasets (14,715 slices), while all 58,860 SwinUNETR slices fail to achieve Dice score (Dice ≤ 80%) (Tables 3 and 4), thereby establishing 3D nnU-Net’s superior performance with regards to cell splitting between overlapping foreground and background image textures.

**Table 2:** Robustness analysis across varying stages of heart maturation

Method	Training data	Day 3 (27,600 slices)	Day 4 (9,900 slices)	Day 5 (11,760 slices)	Day 6 (9,600 slices)	Total slices	Overall performance
3D nnU-Net	full resolution	27,600	9,900	11,760	9,600	58,860	Dice > 90%
3D nnU-Net	25% reduced	6,900	2,475	2,940	2,400	14,715	Dice > 90%
3DeeCellTracker	full resolution	27,600	9,900	11,760	9,600	58,860	Dice ≤ 90%
SwinUNETR	full resolution	27,600	9,900	11,760	9,600	58,860	Dice ≤ 90%

**Table 3:** Cross-day evaluation of 3D nnU-Net

	Day 3	Day 4	Day 5	Day 6
Day 3	0.9608 [0.960,0.962]	0.2923 [0.282,0.302]	0.5980 [0.594,0.602]	0.6581 [0.653,0.663]
Day 4	0.4831 [0.481,0.486]	0.9461 [0.943,0.949]	0.6663 [0.665,0.667]	0.5596 [0.557,0.562]
Day 5	0.1948 [0.193,0.197]	0.2835 [0.272,0.295]	0.9502 [0.949,0.951]	0.4352 [0.432,0.439]
Day 6	0.3802 [0.376,0.384]	0.4844 [0.474,0.495]	0.4968 [0.494,0.500]	0.9734 [0.973,0.974]

Models trained on rows, tested on columns. Values: mean Dice [95% CI]. Yellow cells: within-day performance (Dice > 0.94).

**Table 4:** Cross-day evaluation of SwinUNETR

	Day 3	Day 4	Day 5	Day 6
Day 3	0.8254 [0.822,0.829]	0.5014 [0.497,0.506]	0.2498 [0.248,0.251]	0.6193 [0.616,0.623]
Day 4	0.3716 [0.368,0.375]	0.7276 [0.721,0.734]	0.4074 [0.406,0.409]	0.6459 [0.638,0.654]
Day 5	0.1656 [0.163,0.168]	0.4405 [0.435,0.446]	0.6360 [0.634,0.638]	0.4582 [0.449,0.467]
Day 6	0.3073 [0.304,0.310]	0.5612 [0.555,0.568]	0.3794 [0.378,0.381]	0.7275 [0.713,0.742]

Models trained on rows, tested on columns. Values: mean Dice [95% CI]. Yellow cells: within-day performance (max Dice ≤0.83).

### Evaluation of difference-of-Gaussian blur on 3D nnU-Net model performance

We investigated failure case for nnU-Net model with regards to DoG edge detection (Table 5), by varying Gaussian kernel weights *σ*_1_ and *σ*_2_. Consequently, we generated synthetic 3D + time blobs with varying intensity (2ˆn pixel value = 0 to 65,535 for 16-bit image) to mimic LSM SNR (Figures S3A and S3B). In addition, the model training was conducted on grayscale, nuclei like blob clusters (please see “methods” section), to mimic embryonic cell growth during zebrafish heart maturation (Figures S3C and S3D). Consequently, we analyzed model performance for four test cases based on filter weights computed using *σ*_2_ = $\sqrt{2}$ x *σ*_1_. By subtracting lower order Gaussian kernel image from higher order kernel, we quantified Dice coefficients for local intensity maxima detected at grayscale blob centers. Hence, we analyzed highest Dice coefficients for filter weights corresponding with intensity maxima at object center (refer Table 6, test case 4). On the other hand, test cases 1 and 2 (Table 6) achieved poor blob detection performance (19.0% and 22.0% Dice, respectively). Hence, we hypothesize the corresponding models approximate cell density but fail to localize overlapping blob centers, due to DoG filter weight affecting image texture overlap. Further, we validated the effect of watershed for localizing overlapping features, with regards to poor detection performance if objects are separated by $\frac{1}{2}x\;\left(\text{object}\;\text{diameter}\right)$.

**Table 5:** Cross-day evaluation of 3Dee cell tracker using full resolution data dimensionality

	Day 3	Day 4	Day 5	Day 6
Day 3	0.8860 [0.883,0.889]	0.310 [0.030,0.032]	0.0001 [0.000,0.000]	0.0079 [0.007,0.008]
Day 4	0.3101 [0.300,0.321]	0.6370 [0.616,0.658]	0.0248 [0.022,0.027]	0.5175 [0.501,0.534]
Day 5	0.1311 [0.130,0.132]	0.2007 [0.199,0.203]	0.5417 [0.538,0.546]	0.4167 [0.415,0.419]
Day 6	0.4050 [0.397,0.413]	0.5890 [0.580,0.598]	0.3232 [0.313,0.334]	0.6535 [0.636,0.671]

Models trained on rows, tested on columns. Values: mean Dice [95% CI]. Yellow cells: within-day performance (max Dice ≤ 0.83).

**Table 6:** DoG blur parameter evaluation for nnU-Net segmentation accuracy

ID	Configuration	Volumes generated	Val Dice	Test Dice
Test case 1	*σ*_1_ = 0.5, *σ*_2_ = 0.8, sequential images without watershed	low (20) and high density (20)	0.808	0.190
Test case 2	*σ*_1_ = 1, *σ*_2_ = 1.4, interleaved images without watershed	low (20) and high density (20)	0.825	0.220
Test case 3	*σ*_1_ = 1,*σ*_2_ = 1.4, sequential images without watershed	low (20) and high density (20)	0.923	0.782
Test case 4	*σ*_1_ = 1.5,*σ*_2_ = 2.1, sequential images with watershed	low (20) and high density (20)	0.94	0.91
Test case 5	3-fold average without watershed	low (20) and high density (20)	fold 0: 0.8996 (89.96%) fold 1: 0.8803 (88.03%) fold 2: 0.9177 (91.77%)	0.781

Lastly, to assess if ensemble methods can improve single-fold performance, we trained three independent folds (0, 1, and 2) and averaged their predictions. The 3-folds achieved validation Dice scores of 89.96%, 88.03%, and 91.77%, while test Dice score of 78.14% was essentially identical to the single-fold result of 78.20% (Table 6). Hence, we tested failure cases for the proposed framework with regards to inappropriate scale selection criteria for DoG filter and varying SNR (Figures S3E–S3J).

## Discussion

This study successfully demonstrated and validated a multiscale, cell-specific 3D + time nnU-Net-based architecture for quantifying local cardiac tissue deformation. More importantly, by integrating light sheet, multidimensional imaging pipeline and the automated nnU-Net model, we achieved highly reproducible 4D cell trajectory tracking. Hence, main contribution of the study is an autonomous microscopy platform, capable of localizing cell motion *in vivo* or cell-cycle analysis using time-lapse microscopy. Thereby, avoiding manual intensive feature annotation and enabling biologists to investigate cell proliferation events (mitosis/migration) frame-frame, without being affected by varying SNR or object morphology.

We have also demonstrated the potential of combining single particle tracking linear assignment problem (LAP)^26^ with self-adaptive nnU-Net,^25^ thereby establishing a generalized cell-segmentation and tracking framework across varying spatiotemporal scales.^29^ Further, the implementation of watershed within nnU-Net framework for splitting of binarized labels with high degree of spatial overlap, enhanced depth awareness for instance normalization during feature extraction. This enabled generalization for splitting multidimensional binarized features, characterized by variable SNR and high object density.^30^ Furthermore, DoG preprocessing significantly accelerated our segmentation workflow (68 s per epoch, 250–1,000 epochs) compared to manual segmentation requiring order of days.

Moreover, we demonstrate the scalability of the LAP particle tracking algorithm in the Track Mate Fiji plugin with respect to the complexity of tracking high-order dimensional merged objects without interpolation. Unlike traditional approaches, which often rely on linear data structure, the TrackMate plugin utilizes a graph-based structure to store cell trajectories, allowing accurate linking of higher-dimension merged objects without interpolation. The strict frame-to-frame distant-based linking is particularly effective for managing cell merging time-series events.^29^

With regard to developmental biology insights, we observed anisotropic deformation in different ventricular regions, consistent with previous studies mapping regional cardiac output.^11^ Significantly, no differences in area ratio were quantified between the zebrafish specific 3D nnU-Net output and ground truth nuclei labels segmented using a conventional intensity thresholding approach, thereby validating the application of nnU-Net for high-density cell tracking with variable SNR. In addition, we observed nuclei in the outer curvature exhibiting a more elliptical shape, while nuclei in the inner curvature exhibited less eccentricity, as shown in previous studies. This difference likely reflects regional variations in contractility, as higher contractility in the outer curvature may induce nuclear elongation, while the inner curvature, subjected to lower strain, maintains a more spherical shape. These morphological differences may correspond to the distinct trabecular architecture within the ventricular wall, where the outer curvature, typically containing more prominent trabeculae, experiences greater biomechanical forces compared to the inner curvature.

Previous studies suggest myocardial deformation is essential in the regulation of cardiac muscle maturation and activation of the mechanosensitive signaling pathway such as Notch signaling.^24^ Notably, Notch-responsive *tp1:gfp* reporter activity has been shown to be significantly higher in the outer ventricular region of transgenic *tg(tp1:gfp)* zebrafish,^31^^,^^32^ which aligns with our finding of greater deformation in the outer curvature compared with the atrioventricular region. This suggests that difference in contractility and trabecular structure may influence Notch activation, potentially contributing to cardiac trabeculation. Hence, future studies will correlate myocardial contractility with Notch gene expression to better understand the role of Notch in ventricular trabeculae formation.

Hence, our research demonstrates the efficacy of combining a deep learning platform with automated LSM, for implementing an autonomous image acquisition and segmentation workflow. Thereby, enabling high throughput, trajectory analysis of cell migration events for cell culture *in vitro* or preclinical vertebrate models such as zebrafish *in vivo*. Furthermore, our workflow will tremendously benefit biologists for real-time cell division/proliferations studies or better understanding mechanism of action of bioassays, without prior technical knowledge or parameter tuning. Consequently, benefitting biologists burdened with complex manual feature annotation and improving inter observer reproducibility.

### Limitations of the study

Our study successfully validated the integration of a self-configuring 3D U-Net-based architecture with single particle tracking algorithms, thereby enabling an end-end automated imaging and object detection platform. However, current study was aimed at validating dynamic 3D + time zebrafish nuclei, exhibiting regionally consistent local area deformation and cell sizes. Further research warrants investigating segmentation and cell tracking accuracy with irregular contractility or textures. Furthermore, while current study utilizes the native instance segmentation within 3D nnU-Net, future research scope should explore integration of adaptive instance normalization techniques and impact of varying blur parameter for DoG edge detection. In addition, current study aims at validating fluorescence tomographic images, and hence further investigation for dealing with multimodal imaging data would be beneficial.

## Resource availability

### Lead contact

Further information and requests for resources should be directed to and will be fulfilled by the lead contact, Juhyun Lee (juhyun.lee@uta.edu).

### Materials availability

This study did not generate new materials.

### Data and code availability

• All imaging data reported in the paper will be shared by the lead contact upon request.
• All original code is available in this paper’s supplemental information.
• Any additional information required to reanalyze the data reported in this paper is available from the lead contact upon request.

## Acknowledgments

We are sincerely grateful to the 10.13039/100000002National Institutes of Health (NIH R35GM150947) and the National Research Foundation of Korea (NRF RS-2024-00419286) for supporting our work.

## Author contributions

Writing – original and final draft, T.T., J.-Y.L., and J.L.; writing – review, C.-J.C. and H.K.; hardware and software, T.T., M.D., S.S., P.N., T.-V.L., Y.K., G.-H.J., and Y.D.; supervision and funding acquisition, J.L.

## Declaration of interests

The authors declare no competing interests.

## STAR★Methods

### Key resources table

**Table:** 

REAGENT or RESOURCE	SOURCE	IDENTIFIER
**Chemicals, peptides, and recombinant proteins**		

Tricaine	Sigma-Aldrich	CAS#886-86-2
Low-gelling-temperature agarose	Sigma-Aldrich	CAS#39346-81-1
1-phenyl 2-thiourea	Sigma-Aldrich	CAS#36822-11-4

**Deposited data**		

All imaging data reported in this paper	This paper	Available from lead contact upon request

**Experimental models: Organisms/strains**		

Zebrafish:*Tg(cmlc:GFPnuc)*	UTA IACUC (A17.014).	ZDB-ALT-180717-4

**Software and algorithms**		

Track mate	Ershov et al.^26^	https://doi.org/10.1038/s41592-022-01507-1
nnU-net	Isensee et al.^29^	Isensee et al.^29^
3D Object Counter Plugin	Bolte et al.^32^	https://doi.org/10.1111/j.1365-2818.2006.01706.x
MATLAB (coordinate transformation, area ratio quantification, trajectory visualization)	MathWorks	https://www.mathworks.com
*In vivo* trajectory reconstruction and myocardial deformation analysis	This paper, refer Supplementary code	Teranikar et al.^12^

**Other**		

Home-built Light Sheet Microscope	Teranikar et al.^12^	https://doi.org/10.1016/j.isci.2022.104876

### Experimental model and study participant details

Experiments were performed using transgenic *Tg(cmlc:GFPnuc)* zebrafish embryos obtained from spawning adult zebrafish maintained under the UT Arlington Animal Core Facility and Use Committee (IACUC) protocol (A17.014). Due to cardiac myosin light chain (cmlc) contributing majorly to the heart contractile apparatus, wild-type *Tg(cmlc:GFPnuc)* zebrafish were used to analyze differentiated cardiomyocytes. Sex based differences were not investigated in the current study. Green fluorescent protein is expressed primarily in myocardial nuclei through *cmlc* promoter, enabling wall deformation analysis by visualizing cardiomyocyte trajectories. To suppress pigmentation, 0.0025% 1-phenyl 2-thiourea (Sigma-Aldrich, St-Louis, MO) was introduced to E3 embryonic medium between 20 and 24 h postfertilization. 0.05% tricaine (MS 222, E10521, Sigma-Aldrich, St-Louis, MO) was used to sedate the embryos embedded in 0.5% low-melt agarose gel to avoid sample movement during imaging.^23^ Further, agarose gel was transferred to a Fluorinated Ethylene Propylene (FEP) tube (1677 L, IDEX, Chicago, IL) for sample mounting and refractive index matching between embryos and the surrounding medium (Refractive index of water = 1.33, refractive index of agarose and FEP tube = 1.34).

### Method details

#### Light sheet microscopy (LSM) implementation

Our home-built light sheet microscope features a single-side illumination pathway consisting of a cylindrical lens (LJ1695×RM, Thorlabs) and 4× objective lens (4× Plan Apochromat Plan N, Olympus, Tokyo, Japan). A mechanical slit (VA100C, Thorlabs) was used to vary light sheet thickness, to sample growing ventricular circumference (100–200 μm) across varying developmental stages and anisotropic nuclei sizes (6–12 μm). Optical sectioning of the sample was executed by a DC servo motor actuator (Z825B, Thorlabs), with z-step velocity and acceleration set at 0.005 mm/s. A single color channel detection pathway features a water dipping lens (20×/0.5 NA UMPlanFL N, Olympus, Tokyo, Japan), infinity corrected tube lens (TTL 180-A, Thorlabs), and sCMOS camera (ORCA flash 4.0, Hamamatsu, Japan). The sCMOS Camera [pixel size = 6.5 μm) enabled non-gated 4D (3D + time) cardiac volume acquisition at rapid exposure times between 30 and 50 m s. As the zebrafish ventricle undergoes periodic deformation from peak systole to end-diastole, optical sections were captured at different time points during 4–5 cardiac cycles. In this regard, volumes were reconstructed post hoc to ensure alignment between adjacent optical sections. This process involves estimating the cardiac cycle period via least squares intensity minimization and adjusting relative period shifts to synchronize independent cardiac cycles. In a previous study, we comprehensively discussed the in silico synchronization of 4D volumes as per specific cardiac phases.^24^

#### 3D nnUnet implementation

The nnU-Net v2 was implemented in Python 3.10 using PyTorch v2. Batchgenerators 0.21 was used for data augmentation. Python libraries that were also used include tqdm 4.66.5, dicom2nifti 2.2.10, scikit-image 0.17.2, MedPy 0.4.0, SciPy 1.5.2, batchgenerators 0.21, NumPy 1.19.1, scikit-learn 0.23.2, SimpleITK 1.2.4 and pandas 1.1.1 (See Table 1 for model implementation). For use as a framework, nnU-Net’s source code is available on GitHub (https://github.com/MIC-DKFZ/nnUNet).^29^ Further, we implemented a novel preprocessing approach within nnU-net v2 (Supplemental document S1, https://github.com/JuhyunLeeLab/3D-Zebrafish-nnUNet.git) focusing on the efficiency and accuracy of the inferencing obtained using 3D nnU-Net. Difference of Gaussian (DoG) was integrated with nnUnet v2 preprocessing framework by importing the DoG filter from the SciPy multidimensional image processing library. Edges (zero crossings) of overlapping nuclei at varying depths were localized by altering the blur operator (standard deviation) for the DoG filter.

#### Data acquisition and preprocessing

We acquired 4D LSM greyscale time series image data from embryonic zebrafish across 4–6 dpf across two cardiac cycles. The raw image volumes and training bins were converted to 8-bit format to reduce size and enhance compatibility with deep learning framework. and imported as 512 × 512 ×*m* 8-bit arrays. To standardize input dimensions for neural network, the input of size was defined as A × A × m {A = (2^n^) k |k∈N} where n is the number of levels of network, m is the image depth. This configuration allows for efficient processing during convolutional operation. The input patch sizes for inference were generated using sliding window with half patch size overlap, and output was merged using Gaussian importance weighting to minimize edge artifacts. We utilized a single emission color channel for imaging, and 3 × 3×3 kernel sizes for convolutions during down sampling to capture local spatial features. Furthermore, our preprocessing strategy consisted of Difference-of-Gaussian (DoG) edge detection filter followed by watershed, to effectively separate foreground nuclei from background autofluorescence tissue.

Briefly, the DoG bandpass operation was implemented as follows,

*t*(*x*)∗*g*1(*x*)-*t*(*x*)∗*g*2(*x*) = *t*(*x*)∗(*g*1(*x*)-*g*2(*x*)),^31^ where g1(x) and g2(x) are the Gaussian kernels having varied standard deviations. The grayscale bandpass filtering process involves generating two smoothed versions of the transmission map using different levels of blur and then computing the difference between them. A detailed study regarding effects of blur degree with respect to segmentation accuracy and image processing workflow has been previously published elsewhere.^12^

#### Network architecture and training

Each computational block consisted of sequences of convolutional operations, instance normalization, and Leaky Rectified Linear Unit (LeakyReLU) activation.^25^ We employed Stochastic Gradient Descent (SGD) with Nesterov momentum (*μ* = 0.99) for optimizing network weights, with initial learning rate set to 0.01. To minimize segmentation error, was used a composite loss function comprising Dice loss and Cross Entropy loss functions.^25^ For modeling, we set the default training parameter to 150 epochs, where each epoch consists of 250 iterations. To avoid overfitting, we applied various data augmentation methods, such as rotation, scaling, Gaussian noise, blurring, contrast and brightness adjustment, and gamma correction. The convolutional strides were configured based on input image size, ranging from 1 × 2 × 2 to 2 × 2 × 2, optimizing the network’s adaptability for varying spatial dimensions. The final training process consisted of 1,000 epochs when using larger image batch sizes (m = 120), ensuring robust learning for dataset spanning different stages of ventricular development. Our dataset comprised of 26,000 image slices, providing comprehensive coverage of myocardial cell dynamics.

#### Post-processing output

The trained model generated segmentation output images in NIfTI (.nii) format. To facilitate further analysis and validation, these outputs were converted to TIFF (.tiff) format.

#### Cell counting and trajectory tracking

To perform particle tracking without trajectory splitting or loss between image frames, it is essential to isolate individual binarized nuclei labels from greyscale myocardial nuclei images. To enhance the contrast of nuclei, we applied Difference of Gaussian (DoG) bandpass filtering. In this approach, the n-dimensional grayscale image *I*:*T* = *T*^*n*^ is described by the function Γ_*σ*1,*σ*2_ = *I*∗*G*_*σ*1_-*I*∗*G*_*σ*2_. Here, *G*_*σ*1_ and *G*_*σ*2_ represent Gaussian filters with different standard deviations (*σ*_1_ and *σ*_2_), respectively. The DoG filter is generated by subtracting a Gaussian filtered image with a larger standard deviation from the same image convolved with a narrower deviation. This process effectively highlights feature within a specific frequency range, enhancing the contrast of nuclei. After contrast enhancement, we applied Otsu binarization to separate nuclei from the background. Subsequently, frame-to-frame linking of nuclei trajectories was performed using the linear assignment problem (LAP) framework.^26^^,^^27^^,^^32^

For dynamic nuclei events regarding track branching or nuclei leaving Field-of-View, corresponding nuclei were discarded from further analysis. All time-series nuclei trajectories were manually visualized in MATLAB image coordinate space to inspect missing tracks. Furthermore, LAP Trackmate plugin within Fiji was used to import nuclei trajectories localized using nearest neighborhood interpolation across an entire cardiac cycle.^26^ The algorithm tracks non-branching trajectory segments between adjacent frames for each individual nuclei ID:

- Cost Matrix Construction: The cost matrix for frame-to-frame linking is constructed as an [*n*+*m*]×[*n*+*m*] matrix whereby *n* represents the number of spots in time frame *t* and m are spots in time frame +1. This matrix helps establish a ‘link’ or ‘no-link’ between successive trajectory segments. The cost function estimation across four quadrants to account for linking and non-linking scenarios.
- Linking Cost Calculation: The linking cost is calculated based on the distance between nuclei in consecutive frames. A user-defined maximum distance is imposed. If the calculated distance exceeded the threshold, the cost is set to infinity, indicating no feasible link.
- Non-linking Cost Calculation: The top right [*n*+*m*] and bottom left [*n*+*m*] quadrants of the cost matrix account for segment termination and initiation costs, respectively. These costs are critical when a nucleus, either associated from an existing frame or adjacent optical sections. For cell counting, we utilized the 3D object Counter plugin in ImageJ.^27^

#### Area ratio quantification

A triad of nuclei trajectories spaced between 5 and 15 μm was imported into the MATLAB workspace for single area ratio quantification. In order to avoid any ambiguities for camera perspective and dynamic nuclei displacement, global coordinate vectors in x, y, and z directions are converted into local coordinates using the MATLAB function global2Localcoord().^28^ Briefly, the algorithm workflow consists of calculating centroids (M x 3 vectors) of triangles (polygons) plotted between the triad of global coordinate nuclei vectors along each time step to establish the origin of local coordinate vectors at (0,0,0). Centroids of triangles in local coordinate space were quantified as, $\sum_{i=1}^{n}C_{x}i=\left(G_{x1}i+G_{x2}i+G_{x3}i\right)/3$ , $\sum_{i=1}^{n}C_{y}i=\left(G_{y1}i+G_{y2}i+G_{y3}i\right)/3$, $\sum_{i=1}^{3}C_{z}i=\left(G_{z1}i+G_{z2}i+G_{z3}i\right)/3$ with respect to three corresponding vertices. [*G*_*x*1_*i*,*G*_*y*1_*i*,*G*_*z*1_*i*], [*G*_*X*2_*i*,*G*_*y*2_*i*,*G*_*z*2_*i*], [*G*_*z*3_*i*,*G*_*y*3_*i*,*G*_*z*3_]. For triangle ABC in local coordinate space, area was computed by $\left|\;½\;x\;\right|\;\vec{A}\;x\;\vec{B}+\vec{B}\;x\;\vec{C}+\vec{A}\;x\;\vec{C}|\;$^11^^,^^12^

#### Synthetic data generation for ablation analysis

Synthetic blob images were generated as 3D grayscale stacks (16-bit depth) with controlled density levels: high and low density using Python. *n* = 160 2Dslices were generated per 3D volume (20 volumes per density).The synthetic images were injected with random SNR (1–7), with pixel spatial distributions resembling zebrafish embryonic nuclei occlusions.

### Quantification and statistical analysis

We generated performance metrics, including line graphs depicting training loss, testing loss, and the Dice coefficient over 1,000 epochs. Dice coefficient was calculated as 2 TP/(2 TP + FP + FN), and Jaccard Index/Intersection of union was calculated as TP/(TP + FP + FN). (TP = true positive, FP = false positive, FN = false negative). One-way ANOVA was used to quantify statistical significance between nuclei counts at multiple heart maturation stages. In case of significant difference for any comparison (*p* value ≤0.5), we performed Tukey’s test for multiple comparison of means. All values in the manuscript or figures (Figures 4 and 5) are reported as mean plus or minus standard deviation.

## Supplemental information

Document S1. Figures S1–S3 and Method S1

Data S1. MATLAB code to convert nuclei coordinates from global pixel coordinates to local coordinate system

Data S2. MATLAB code to quantify area ratio based on area of triangle using pixel local coordinate system

Data S3. MATLAB code to visualize nuclei displacement vectors

Data S4. MATLAB code to generate GIF of nuclei displacement vectors
