# Supplementary material for: Development of a 3D nnU-Net-based cell tracking platform for quantifying myocardial deformation in zebrafish
Source: iScience. 2026 Jun 18;29(7):116376. doi: 10.1016/j.isci.2026.116376 (PMC13312083; doi:10.1016/j.isci.2026.116376)
Supplement: Document S1. Figures S1–S3 and Method S1 [file mmc1.pdf]

iScience, Volume *29*

Supplemental information

Development of a 3D nnU-Net-based cell tracking platform for quantifying myocardial deformation in zebrafish

Tanveer Teranikar, Mishu Devadasan, The Van Le, Yoonsuk Kang, Gilberto Hernandez Jr., Phuc Nguyen, Yichen Ding, Cheng-Jen Chuong, Jin Young Lee, Hyunsuk Ko, and Juhyun Lee

SUPPLEMENTAL FIGURES

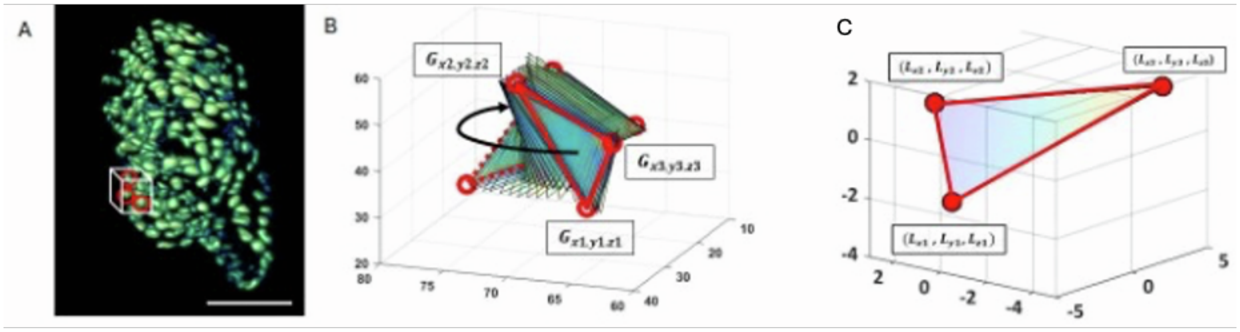

Supplemental Figure 1. Quantifying area ratio using cardiomyocyte trajectories acquired in vivo, (A) 6dpf myocardial nuclei in zebrafish ventricle. A nuclei triad is required to compute area of triangle for mechanical deformation (scale bar = 50 μm). (B) Triangle (polygon) plotting was performed in a global coordinate system across the entire cardiac cycle to quantify centroids required for origin. Solid red boundary indicates systole, dotted red boundary indicates diastole. The grid used for visualization represents pixel coordinates (C) Nuclei position vectors reconstructed in local coordinate space for a single image frame, centered at (0,0,0) to avoid inaccuracies in area quantification due to camera perspective and nuclei displacement. The triangle reconstructed between nuclei biomarkers is reconstructed in a local coordinate system inferred from global pixel coordinate system in (B).

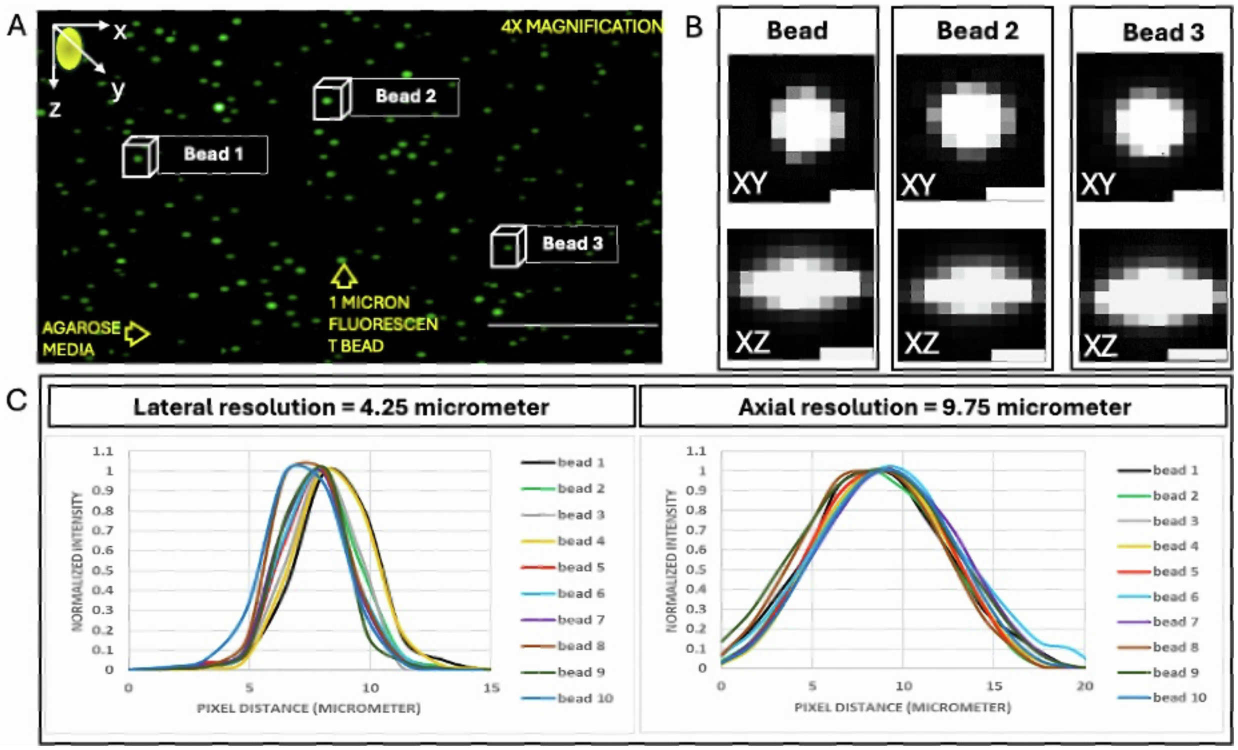

Supplemental Figure 2. Quantification of light sheet system resolution. (A)500 micrometer field-of-view produced by 4x lens. (B)Beads (n=10) inspected at random across the FOV for resolution quantification. (scale bar for B = 4 micron). (C) Gaussian intensity profiles used for calculation of full width half maximum.

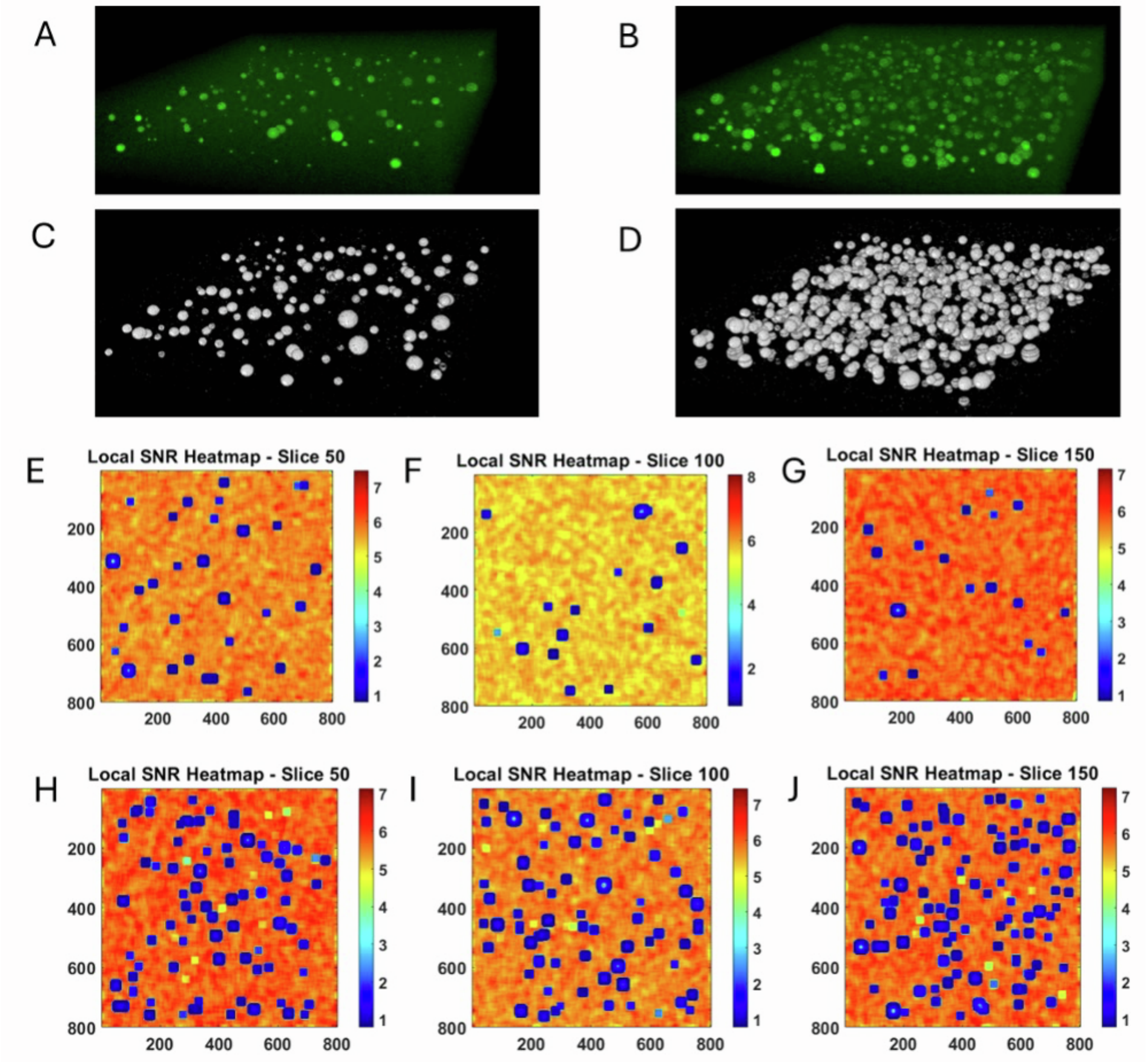

Supplemental Figure 3. Assessment of DoG filter for varying SNR using synthetic data. (A), (B) represent low and high density, synthetic greyscale datasets with varying SNR generated using Python. (C) and (D) represent binarized objects using DoG $\sigma_1 = 1$, σ_2 = 1.4 without watershed for ablation analysis. (E) – (G) represents the filter sensitivity with respect to random noise represented by heat map for low density volume (H) – (J) represent filter sensitivity for high-density binary cluster of overlapping features.

Method S1. Step-by-step implementation guide for 3D nnU-Net environment setup and MATLAB analysis scripts. (related to STAR Methods).

Cell splitting based nnU-net

The supplementary document describes a comprehensive segmentation workflow utilizing nnUnet (https://github.com/JuhyunLeeLab/3D-Zebrafish-nnUNet.git), based on unique features of a dataset (image size, voxel data etc). The segmentation pipeline includes data preparation, model setup, training, result processing, in addition to combining multiple models (2D, 3D modes). After executing nnU-Net, the resulting model(s) can be utilized for inference on test data. In addition, we have implemented an alternate cardiac

Setting up the environment

1. Create new environment.
    a. Enter command: conda create -n NAME python==3.10
        i. NAME is desired environment name. Ex: nn_UNet
        ii. conda create -n nn_UNet python==3.10

2. Activate environment:
    a. conda activate NAME
        i. Ex: conda activate nn_UNet
            1. Check version. Enter command: python –version

3. Cd to the directory where all the zebrafish repositories will be stored.

4. Enter command **git clone https://github.com/nasyxx/zebrafish_seg.git**
    a. If git is not installed then – **sudo apt install git – all**
    b. Ex: **conda install pytorch torchvision torchaudio pytorch-cuda=11.8 -c pytorch -c nvidia**

5. Transfer the tiff_converter.py file into this directory.

6. cd into *data* directory

    c. Enter command: **mkdir raw ori segmented results preprocessed cropped inference**

7. Save your environment variables.

    a. Enter command: **vim ~/.bashrc**

        i. If not installed enter following command: **sudo apt install vim**

            1. Retry command after installation is finished

        ii. If blank, double check spelling and retry.

        iii. Press Insert key to begin editing.

    b. Move indicator to very bottom of page and add:

export nnUNet_raw_data_base=data/raw/

export nnUNet_raw=data/raw/nnUNet_raw_data

export nnUNet_results=data/results/

export nnUNet_preprocessed=data/preprocessed/

export RESULTS_FOLDER=/home/USER/nnUNetFrame/zebrafish_seg/data/results/

    c. Press Escape key to return to command mode.

            1. Enter **:x** to save and exit (Alternatively enter **:wq**)

            2. Enter **:q!** to exit WITHOUT saving

8. Source your environmental variables:

    d. Enter command: **source ~/.bashrc**

e. Reactivate your environment: **conda activate NAME**

i. Ex: **conda activate nn_UNet**

9. Install nnUNet repository

d. **git clone https://github.com/MIC-DKFZ/nnUNet.git**

e. **cd nnUNet**

f. **pip install -e .**

g. **pip install --upgrade git+https://github.com/FabianIsensee/hiddenlayer.git**

10. Install pytorch https://pytorch.org/get-started/locally/

h. Follow directions to install according to computer specifications

11. Install pdm and start project

i. **python -m pip install pdm**

j. **pdm init**

Follow the prompts. You will see:

"Please enter the Python interpreter to use

0. /home/leelab/anaconda3/envs/zebrafish_seg/bin/python (3.10)

1. /home/leelab/anaconda3/envs/zebrafish_seg/bin/python3.10 (3.10)

2. /usr/bin/python3.10 (3.10)

Please select (0): "

Enter whichever is your preference. Ex: *Please select (0):* 2

"Is the project a library that is installable?

If yes, we will need to ask a few more questions to include the project name and build backend [y/n] (n):"

Enter: **y**

Project name: **Zebrafish Segmentation**

Project version (0.1.0): **0.1.0**

Project description (): **Auto-segmentation tool**

"Which build backend to use?

0. pdm-backend

1. Setuptools

2. flit-core

3. Hatchling "

Enter: **0**

"License (SPDX name) (MIT): **AFL-1.1**

Author name (): **JL**

Author email (): juhyun.lee@uta.edu

Python requires('' to allow any) (>=3.10):* **>=3.10**"

12. Install remaining dependencies:

    k. **pdm run pip install tqdm**

    l. **pdm run pip install pathlib**

    m. **pdm run pip install rich**

    n. **pdm run pip install smile_config**

    o. **pdm run pip install tifffile**

    p. **pdm run pip install nnunet**

    q. **pdm run pip install nnunetv2**

       i. Note, some may not install. Keep an eye on errors for future commands to see which libraries are missing.

Dataset Conversion

13. Cd to *src* folder, then open dc.py

    f. Either with command: **vim dc.py**

        i. Press Insert key to begin editing

        ii. If not installed enter following command: **sudo apt install vim**

            1. Try again when installed

    g. Or may be opened and edited using Notepad

Edit line 75:

    h. **Space = tuple(map(float,conf.space.split(","")))**

14. Comment out lines 88, 100-119 using #

    i. If on vim, press Escape key to return to command mode

        i. Use command **:x** or **:wq** to save and exit, note to enter commands the command must begin with colon key " **:** "

        ii. Use command **:q!** to exit WITHOUT saving

    j. If on Notepad, simply save the file before exiting.

        i. Either CTRL + S

        ii. On menu bar File>Save

15. Return to directory containing zebrafish_seg repositories from github

    k. Enter in terminal while in *src* directory: **cd ..**

16. Enter command: **pdm run python -m src.dc --tod --ori path/to/raw --seg path/to/seg --database path/to/database_directory --name NAME –t XXX**

    l. Note:

        i. If environment variables are set up already, you do not need to specify --ori, --seg, --database.

        ii. path/to/raw is the pathway to the ori directory. Default: data/ori

iii. path/to/seg is the pathway to the segmented directory. Default: data/segmented

iv. path/to/database_directory is pathway to zebrafish database. Default: data/

v. NAME is to be replaced with desired name. Ex: zebrafish_segv2

vi. XXX is to be replaced with three digit task identifier number. Ex: 777

17. Convert Task (nnUNetv1 format) to Dataset (nnUNetv2 format):

    m. **pdm run nnUNetv2_convert_old_nnUNet_dataset Path/To/Task/Folder DatasetXXX_NAME**

        i. Path/To/Task/Folder is the pathway to where the task is located. Ex: data/raw/nnUNet_raw_data/Task777_zebrafish_segv2

        ii. Replace XXX with three digit dataset identification number. Ex: 777

        iii. Replace NAME with desired name for the dataset. Ex: zebrafish_segv2

            1. Final product example: Dataset777_zebrafish_segv2

Plan and Preprocess

1. Edit default preprocessor to include Laplacian of Gaussian.

    a. Download default_preprocessor.py file containing the pre-added code. Move to directory containing the nnUNet repositories. Default preprocessor can be found at: *nnUNet* > *nnUNetv2* > *preprocessing* > *preprocessors* > *default_preprocessor.py*

        i. Delete default_preprocessor.py and replace with the updated default_preprocessor.py file. The new version will include the Lapacian of Gaussian code using scipy library.

2. Begin plan and preprocessing.

    a. Enter code:

    pdm run nnUNetv2_plan_and_preprocess -d XXX -c 3d_fullres --verify_dataset_integrity

    b. Replace XXX with Dataset three digit ID. Ex: pdm run nnUNetv2_plan_and_preprocess -d 777 -c 3d_fullres --verify_dataset_integrity

3. (Optional) To check to see if the new preprocessor is working as intended, utilize the tiff_converter.py file.
    a. Make new directory in data that will contain the preprocessed .tif files.
        i. (Optional) Create subdirectory for the converted .npz to .tif files for organizational purposes.
    b. Enter command: **vim tiff_converter.py**
    c. Replace input_folder = ‘path/to/preprocessed/images’
        i. Ex. input_folder = '/home/senior/nnUNetFrame/zebrafish_seg/data/preprocessed/Dataset444_zebrafish/nnUNetPlans_3d_fullres/'
    d. Replace output_folder= ’path/to/tiff/output’
        i. Ex: output_folder = '/home/senior/nnUNetFrame/zebrafish_seg/data/tiff_files/Dataset444_zebrafish_seg'
    e. Enter command: **pdm run python tiff_converter.py**

4. Once completed, begin training.
    a. Enter code**: nnUNetv2_train DATASET_NAME_OR_ID 3d_fullres 5**
        i. Replace DATASET_NAME_OR_ID with dataset id used for plan and preprocessing. Ex: pdm run nnUNetv2_train 777 3d_fullres 5
        ii. Results will be found in data/results

Analysis and Visualization of 3D Nuclei Displacement and Area Dynamics

This repository contains MATLAB scripts used for processing, analyzing, and visualizing the displacement and area dynamics of cell nuclei over time from 3D volumetric TIFF datasets. These scripts support the generation of quantitative data and visual outputs such as centroid displacement fields, area ratios, and animated 3D representations.

arearatio.m (refer Data S2)

Purpose

Computes and visualizes area ratios of 3-point nuclei triangles over time using geometric cross-products.

Inputs

- Excel file with nuclei coordinates ; Each row should contain 3 nuclei with X, Y, Z coordinates for each point.

Outputs

- 3D plots of triangles at each frame
- Calculated area for each triangle
- **Smoothed and normalized area ratio time series**

Workflow

1. Read Excel file and remove missing values
2. Construct 3D triangles from 3 nuclei per row
3. Compute triangle area using vector cross products

4. Normalize and smooth the area values

5. Generate plots:

    - Raw area values

    - Smoothed and normalized area ratios

    - Optional: plot against a time axis (e.g., milliseconds)

displacementvector.m (refer Data S3)

Purpose

Loads a time series of 3D TIFF image stacks, extracts centroids of segmented nuclei, and visualizes their displacement between time points using deformation fields.

Inputs

- **TIFF files** in subfolders located at a user-specified path (BaseDir)

- numOfSlices: Number of z-slices per volume (e.g., 184)

- numOfFolders: Number of time points (e.g., 120)

- **User selections** for centroid-based triangle plotting and vector visualization

Outputs

- 3D scatter plots of nuclei centroids

- Two user-defined triangular surfaces

- **Displacement vectors** overlaid on triangle centroids using imregdemons

Workflow

1. Load a 4D volume (time-series of 3D TIFFs)
2. Binarize and extract 3D centroids for each time point
3. User selects two triangles interactively from a specified time point
4. Compute displacement field between two time-adjacent 3D volumes
5. Visualize vectors showing nuclei displacement across the triangles

GIFcreationglobalcoordinates.m (refer Data S4)

Purpose

Creates a GIF animation of all triangles formed from nuclei coordinates over time in 3D space.

Inputs

- Excel file with the same structure as in arearatio.m

Outputs

- GIF file (all_triangles_held1.gif) showing 3D evolution of triangle configurations over time

Workflow

1. Read nuclei coordinate data
2. Plot semi-transparent triangles in a 3D space
3. Append each triangle as a frame into a GIF file

Notes and Recommendations

- Ensure the Excel coordinate files match expected formats:

  Each row must contain coordinates of 3 nuclei (X, Y, Z for each).

- You may need the smoothdata2 function for smoothing operations.

- Use consistent file paths or update them as needed (BaseDir, Excel paths).

- MATLAB Image Processing Toolbox is required for functions like regionprops3, imregdemons.
